# Spiny mice (*Acomys*) cells fail to engraft in NOD scid gamma

**DOI:** 10.1371/journal.pone.0286000

**Published:** 2023-05-19

**Authors:** Janak Gaire, Valentina Supper, Darrice Montgomery, Chelsey S. Simmons

**Affiliations:** 1 Department of Mechanical & Aerospace Engineering, University of Florida, Gainesville, Florida, United States of America; 2 J. Crayton Pruitt Family Department of Biomedical Engineering, University of Florida, Gainesville, Florida, United States of America; University of Minnesota Medical School, UNITED STATES

## Abstract

Immune cells and stromal cells regulate wound healing and regeneration through complex activation patterns with spatiotemporal variation. The scarless regeneration of Spiny mice (*Acomys* species) is no exception; differential activation of immune and stromal cell populations seems to play a role in its remarkable regenerative capacity. To elucidate the role and interplay of Acomys immune cells in mammalian regeneration, we sought to create *Acomys*-*Mus* chimeras by transplanting bone marrow (BM) from *Acomys* into NOD Scid Gamma (NSG), a severely immunodeficient mouse line often used in creating humanized mice. Here, we report that *Acomys* BM cells fail to reconstitute and differentiate when transferred to irradiated NSG adults and neonates. In addition, we did not detect the presence of donor cells nor observe the onset of Graft versus Host Disease (GvHD)-like pathology, even after transplanting *Acomys* splenocytes in *Acomys*-*Mus* chimeras suggesting early graft failure. Overall, these results demonstrate the adoptive transfer of *Acomys* BM alone is not sufficient to establish *Acomys* hematopoietic system in NSG mouse.

## Introduction

Spiny mice *(Acomys* species) are an emerging research organism in the field of regenerative medicine that can regenerate a wide range of tissues after injury [1–3]. Multiple species of *Acomys* have been demonstrated to fully replace large patches of dermal wounds and ear biopsy punches without scarring [4–6], and there are also indications of its ability to regenerate internal tissues, including skeletal muscle [7], nervous [8, 9], renal [10], and cardiovascular tissues [11–13] with minimal fibrosis. As such, *Acomys* presents researchers with the opportunity to study the regenerative phenomenon in adult mammals in the hopes of understanding and fixing many fibrotic conditions.

Though the underlying biological mechanisms are unclear, previous studies indicate that altered activation of immune cells and stromal cells (i.e., fibroblasts) likely contribute to scarless regeneration in *Acomys*. A dampened immune response is observed at the *Acomys* wound bed, as suggested by the reduced pro-inflammatory activity [14–16]. Regeneration is associated with the blunted immune response not only in *Acomys* but also in other traditional regenerative models such as salamanders, zebrafish, and fetal mammalian tissues [17–19]. Interestingly, fibroblasts from regenerative *Acomys* also remodel collagen differently than scar-forming counterpart, *Mus*. *Acomys* fibroblasts cultured on stiff substrates neither turn into myofibroblast phenotype [20] nor deposit excessive collagen *in vivo* [14], suggesting differential activation of fibroblasts may be involved in maintaining a pro-regenerative niche.

It is well established that the activity of immune and stromal cells tightly regulates regeneration and fibrosis; however, the cause-and-effect relationship between these cell types is not fully understood. To this end, we sought to create *Acomys-Mus* chimeras (“*Acomyzed* mice”) by transferring bone marrow (BM), as a source of hematopoietic stem cells (HSCs), from adult *Acomys* into NOD *scid* gamma (NSG) mice. NSG mice are severely immunodeficient, lacking an adaptive immune component (T and B cells) and demonstrating impaired natural killer cell activity [21]. For this reason, they are popular hosts for tissue xenografts, and indeed, have resulted in chimeras after xenogeneic transplants from humans [22–26] and bats [27]. Ideally, *Acomyzed* mice would have the immune cells of regenerative *Acomys* and the stromal component (i.e., fibroblasts) of *Mus*, a non-regenerative counterpart that heals by forming a scar. Thus, engineered chimeras would permit investigations on the role of *Acomys* immune cells and their causal relationship with stromal cells in mammalian regeneration and fibrosis across a wide range of injury models and pro-fibrotic conditions.

## Materials and methods

### Animals

All experimental procedures involved in this study were in accordance with United States Department of Agriculture (USDA) and National Institute of Health (NIH) guidelines and were approved by the Institutional Animal Care and Usage Committee (IACUC) at the University of Florida (Protocol Number: 202110302). Spiny mice (*Acomys cahirinus*) were bred at the University of Florida, CD-1 strain of the laboratory mice (*Mus musculus*) were purchased from Charles River Laboratories (Wilmington, MA), and NOD *scid* gamma (NSG) (Stock No. 005557) were purchased from The Jackson Laboratory (Bar Harbor, ME) and bred in the animal facility at the University of Florida.

Chimeras were generated *via* adoptive transfer of *Acomys* and *Mus* cells (BM and splenocytes) into NSG recipients using protocols adapted from humanized mice [26, 28] and bat-mice [27]. In brief, adult NSGs (6–12 weeks old) and NSG pups (postnatal ≤ 3 days) were sub-lethally irradiated with a dose of 240 rads and 100 rads, respectively using an X-ray irradiator (MultiRad 350, Precision X-ray). Between 4–24 hours post-irradiation, freshly prepared donor cells (1–2 X 10^6 cells/recipient) or sterile saline were transferred to adult and neonatal NSGs *via* intravenous (tail vein) and intra-hepatic route, respectively. Irradiated NSG recipients that received sterile saline and *Mus* cells served as negative and positive controls, respectively.

### Bone marrow (BM) preparation

BM from male donors (*Acomys* and *Mus*) was used as a source of HSCs. Donor animals were euthanized by CO2 asphyxiation and long bones (tibia and femurs) were harvested, and placed in ice-cold, sterile phosphate-buffered solution (PBS) containing 2% fetal bovine serum (FBS). After isolation, cells were resuspended in PBS containing 2% FBS. Red blood cells (RBCs) were lysed using 1X RBC lysis buffer (BD Pharm Lyse, Catalog# 555899) and washed with PBS containing 2% FBS. Cells were then passed through a 40 μm mesh filter (Fisherbrand, catalog # 22363547) to remove clumps and assessed for viability using trypan blue. Cells were kept on ice until transfer.

### Splenocyte preparation

Immediately after CO2-induced euthanasia, the spleen was harvested and kept on ice-cold RPMI media containing 5% FBS. Spleen was dissociated into single cells using sterile frosted slides and passed through a 70 μm nylon mesh filter (Fisherbrand, catalog # 22363548) to remove debris. RBCs were lysed using RBC lysis buffer (BD Pharm Lyse, Catalog# 555899) for 5 mins and washed with ice-cold media. Cells were resuspended in sterile PBS containing 2% FBS and passed through a 40 μm nylon mesh filter (Fisherbrand, catalog # 22363547) to remove cell clumps. Filtered cells were assessed for viability and counted. Cells were kept on ice until transfer or staining.

### DNA isolation and polymerase chain reaction (PCR)

Peripheral blood was collected from the facial vein (longitudinal) or trunk (endpoint) and stored at -80°C. DNA was isolated using QIAamp DNA Micro Kit (Qiagen, catalog # 56304) following the manufacturer’s recommended guidelines. DNA quality and quantity were determined using a plate reader (BioTek Synergy H1, Agilent Technologies) and PCR was performed using species-specific primers as shown in Table 1. The PCR products were loaded on agarose gels (E-Gel^™^ double comb agarose gels with SYBRTM safe DNA gel, Invitrogen by ThermoFisher Scientific, catalog # A42348) and ran for 10 minutes (E-gel Power Snap, Invitrogen by ThermoFisher Scientific). Images were acquired using a LI-COR imaging system (LI-COR Odyssey Fc, Lincoln, NE).

**Table 1:** Species-specific primers.

Target	Species	Forward primer	Reverse primer	Size
**CD45 (pan leukocyte)**	*Acomys*	CATATACGGTTGGCTATGGCTAC	TCCGCATGTTGAGTGGATTTATAC	656
**SrY (Y chromosome)**	*Acomys*	TCAGCAAGCTGTTAGGATACCA	CCTGCGACGAGGTTGATATT	134
	*Mus*	CCAGGAGGCACAGAGATTGA	GTGCAGCTCTACTCCAGTCT	200

### Flow cytometry

Freshly prepared splenocytes were incubated in mouse Fc block (Rat anti-mouse CD16/32, BD Biosciences, catalog # 553142, 1:100) for 5 mins followed by incubation in antibodies for pan-leukocytes (Alexa Fluor 647 anti-mouse CD45, BioLegend, catalog # 103124, 1:100), B cells (PE-Cy7 Rat anti-mouse CD45R/B220, BD Biosciences, catalog # 552772, 1:100), and T cells (BV605 anti-mouse CD3, BioLegend, catalog # 100237, 1:100) in dark at 4°C for 30–45 mins, washed with PBS containing 2% FBS. Cells were stored on ice until analyzed using cytometer (CYTEK Northern Lights 3000, Fremont, CA).

### Histology

After CO2-induced euthanasia, multiple organs (spleen, liver, and kidney) were collected and fixed in 4% paraformaldehyde for 24 hours and processed for paraffin embedding. Tissues embedded in paraffin blocks were sectioned using a microtome (Microm HM355S, Thermo Scientific) and transferred to glass slides (FisherbrandTM Superfrost plus, Pittsburgh, PA). Tissue sections were stained with H&E solution (Hematoxylin: GHS280, Sigma Aldrich; Eosin: HT1101128, Sigma Aldrich) and imaged using a Keyence microscope (BZX-100). Tissues were analyzed by observing for splenic clustering in the white pulp regions of the spleen associated with follicular development of lymphocytes [29] and infiltration of mononuclear cells in the kidneys and livers associated with GVHD-like pathology [30, 31].

## Results

### Tail-vein injection of *Acomys* BM in NSG adults did not result in reconstitution of *Acomys* immune system

**Fig 1:**
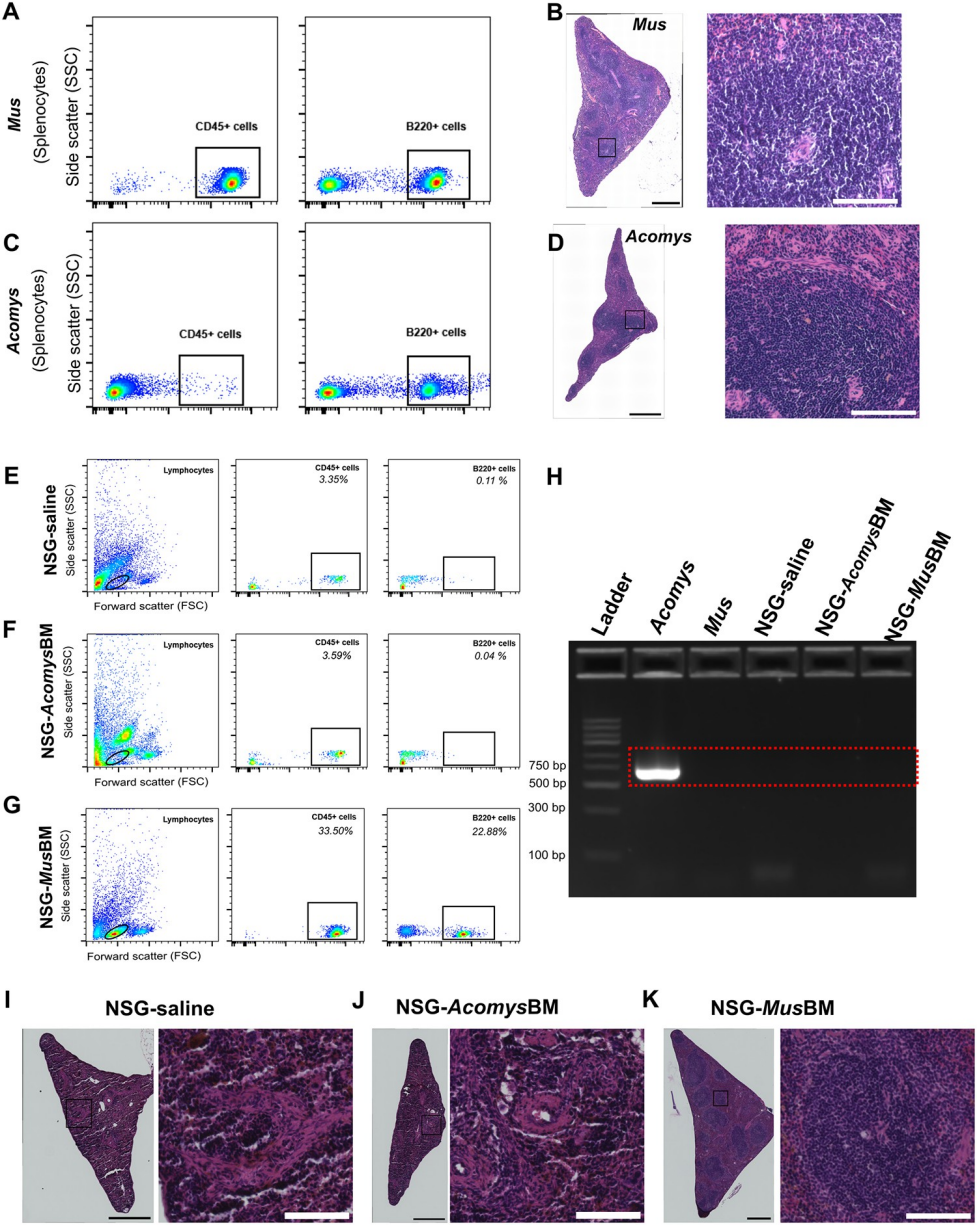
Tail-vein injection of *Acomys* bone marrow did not lead to reconstitution of *Acomys* immune system in adult NSGs. (A-D) Representative flow cytometry and H&E-stained spleen sections from *Mus* and *Acomys*. Splenocytes stained with CD45, a pan leukocyte marker and B220, a B cell marker from *Mus* (A) and *Acomys* (C) demonstrating commercially available CD45 antibody fail to cross-react with *Acomys* cells. Clusters of mononucleated lymphocytes are present in the white pulp region of spleen in immunocompetent *Mus* (B) and *Acomys* (D). (E-G) Representative results of screening for chimerism in spleen from different groups demonstrating absence of CD45+ and B220+ cells in NSG-saline groups (E) as expected, absence of B220+ cells (note CD45 does not cross react with *Acomys* cells) in NSG-*Acomys* BM (F), and presence of CD45+ and B220+ cells in NSG-*Mus* BM groups (G). H. Screening of chimerism from DNA extracted from facial bleed using *Acomys*-specific CD45 primers confirming the failure of reconstitution and maturation of *Acomys* immune system in *Acomyzed* mice. (I-K) Representative images of H&E stained spleen sections from NSG-saline (I), NSG-*Acomys*BM (J), NSG-*Mus*BM (K). Absence of clusters of mononucleated cells in NSG-*Acomys* BM further confirms no reconstitution and maturation of *Acomys* immune system. Clusters of mononucleated cells are present in the spleen of NSG-*Mus*BM. Scale bars in stitched images (B, D, & I-K) are 500 μm. Scale bars in enlarged images are 100 μm.

To create *Acomyzed* mice, BM from *Acomys* was adoptively transferred to irradiated adult NSGs *via* tail vein. Irradiated NSG mice injected with saline (NSG-saline) and BM from CD1 strain *Mus* (NSG-*MusBM*) served as negative and positive controls, respectively. Given the novelty of *Acomys* in regenerative medicine and previous reports on the lack of immunoreactivity of certain antibodies [32, 33], we first validated the immunoreactivity of commercially available antibodies for pan-leukocytes (CD45) and B cells (B220). Consistent with previous reports, we found that CD45 failed to cross-react with *Acomys* lymphocytes whereas B220 identified B cells in *Acomys* spleen (Fig 1A and 1C). As CD45 antibody did not show reactivity with *Acomys* cells, we relied on B cell marker that is absent on NSG mice and cross-reacts with *Acomys* B cells for screening chimeras. In addition, we designed *Acomys* specific CD45 primers (Table 1) to aid in longitudinal screening for chimeras using peripheral blood.

Starting 8 weeks post-transfer, blood was collected from the facial vein to longitudinally screen for the presence of donor cells using flow cytometry or polymerase chain reaction (PCR). In our PCR experiments, conducted using DNA isolated from blood collected via facial vein, we did not detect the presence of *Acomys* CD45 cells in NSG-*Acomys*BM animals at 8 weeks post-transfer (Fig 1H) and thereafter. After 4+ months post-transfer, animals were euthanized, and spleens were harvested to screen for chimerism using flow cytometry and histology. Since NSG mice lack T and B cells, we did not observe any CD45+ and B220+ cells in the splenocytes of NSG-saline groups (Fig 1E) as expected. When gated for lymphocytes based on size (i.e., side- and forward-scatter properties alone), the lymphocyte populations between NSG-saline and NSG-*Acomys*BM groups were comparable (Fig 1E and 1F). We did not observe any B220+ cells in the splenocytes of NSG-*Acomys*BM groups via flow nor histology, indicating *Acomys* HSCs did not differentiate into B cells and migrate to the spleen of NSG recipients. As expected, hypoplasia was observed in NSG-saline animals and indicated by the absence of mononucleated clusters of lymphocytes in the white pulp regions of the spleen (Fig 1I). The spleens of NSG-*Acomys*BM groups (Fig 1J) were similar to that of NSG-saline groups (Fig 1I), further corroborating the failure of *Acomys* HSCs to engraft, differentiate, and mature into lymphocytes that populate the spleen. On the other hand, BM from immunocompetent *Mus* reconstituted and differentiated into mature lymphocytes as indicated by the presence of CD45+ lymphocytes and B220+ B cells (Fig 1G). Furthermore, clusters of mononucleated cells populating the white pulp regions of the spleen were observed in NSG-*Mus*BM group (Fig 1K), a phenotype similar to the organization of spleen as seen in immunocompetent *Mus* (Fig 1B) and *Acomys* (Fig 1D). Overall, following BM injection in irradiated adult NSG mice, we did not find signs of chimerism in any NSG-*Acomys*BM animals (n = 11), whereas all NSG-*Mus*BM (n = 9) successfully reconstituted, differentiated, and populated to peripheral blood and lymphoid organs (i.e., spleen).

### Intrahepatic delivery of *Acomys* bone marrow in NSG neonates did not result in reconstitution of *Acomys* immune system

**Fig 2:**
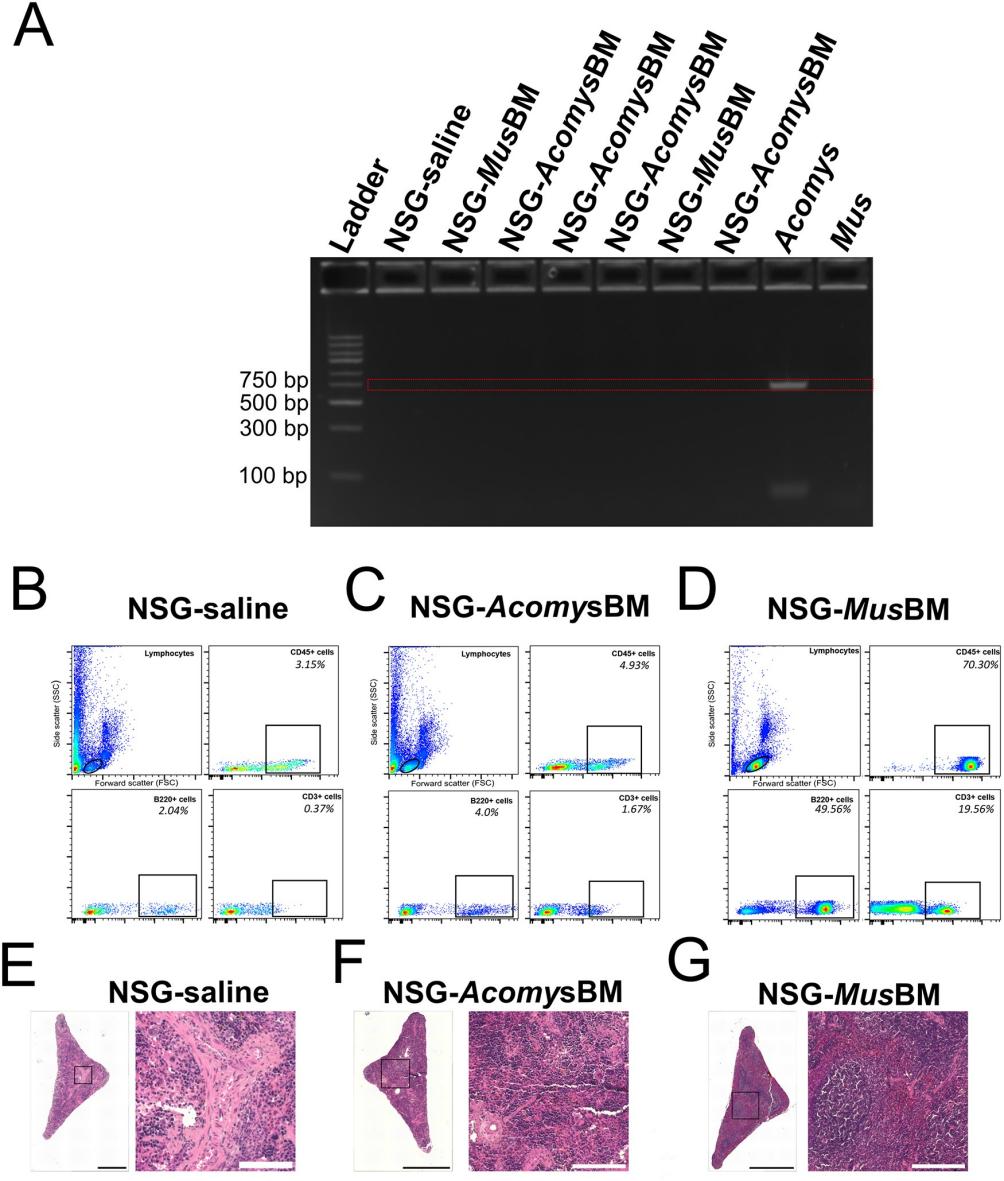
Intra-hepatic delivery of *Acomys* BM did not lead to reconstitution of *Acomys* immune system in neonatal NSGs. (A) Representative result of screening for chimerism *via* PCR using *Acomys* specific CD45 primers at 8 weeks post-transfer. *Acomys*-specific CD45+ cells were absent in the peripheral blood of NSG-*Acomys*BM groups. (B-D) Flow cytometry results of splenocytes from different groups stained with CD45 for pan leukocytes, B220 for B cells and CD3 for T cell at 16+ weeks post-transfer. NSG-saline groups lack CD45+, B220+, and CD3+ cells as expected. Absence of B220+ and CD3+ cells in NSG-*Acomys*BM suggests failure of Acomys BM to reconstitute, mature and populate the spleen. The presence of CD45+, B220+, and CD3+ cells in NSG-*Mus*BM demonstrates successful reconstitution, maturation, and distribution of donor HSCs in immunodeficient NSG mice. (E-G) Representative images of H&E-stained spleen from NSG-saline (E), NSG-*Acomys*BM (F), and NSG-*Mus*BM (G) at 16+ weeks post transfer. Absence of clusters of mononucleated cells in NSG-*Acomys* BM further confirms no reconstitution and maturation of *Acomys* immune system in recipient animals. Clusters of mononucleated cells are present in the spleen of NSG-*Mus* BM. Scale bars in stitched images are 500 μm. Scale bars in enlarged images are 100 μm.

The failure to reconstitute *Acomys* immune system in NSG adults could be due to the rapid clearance of transplanted BM from the periphery. We hypothesized that engraftment could be improved by delivering *Acomys* BM into the liver of NSG neonates since extensive expansion of HSCs naturally occurs in the peri-natal liver. Furthermore, there are also reports of success in generating humanized mice with a more developed human immune system using neonates as a host [26, 34, 35]. Accordingly, we conducted the next set of experiments by injecting *Acomys* BM into the livers of NSG neonates (postnatal 2–3 days, n = 9). We again included NSG-*Mus*BM (n = 6) and NSG-saline groups (n = 6) as positive and negative controls, respectively. Starting at 8 weeks post-transfer, we screened for chimerism using *Acomys* specific CD45 primers and flow cytometry. Similar to tail-vein experiments, we did not observe substantial amount of CD45+ cells in the DNA isolated from peripheral blood of NSG-*Acomys*BM chimeras at 8 weeks (Fig 2A) and beyond. At 16+ weeks, animals were euthanized, and spleens were harvested to screen for chimerism using flow cytometry and histology. Similar to results from tail-vein experiments in adult recipients, screening results from NSG-*Acomys*BM groups were comparable to NSG-saline groups (n = 6). Splenocytes gated for lymphocytes in all animals from NSG-*Acomys*BM groups were neither positive for B cells nor T cells as indicated by the absence of B220+ and CD3+ cells, respectively (Fig 2B and 2C). The spleens of NSG-saline and NSG-*Acomys*BM groups look identical with no evidence of splenic clustering in the white pulp regions (Fig 2E and 2F). In contrast, splenocytes from animals in NSG-*Mus* BM groups were positive for CD45 (pan leukocytes), B220 (B cells), and CD3 (T cells) (Fig 2D) and splenic clusters of mononucleated cells were present in the white pulp regions of 5 out of 6 animals in NSG-*Mus*BM group (Fig 2G).

### *Acomys* cells fail to engraft and trigger GvHD-like pathology in NSG host

**Fig 3:**
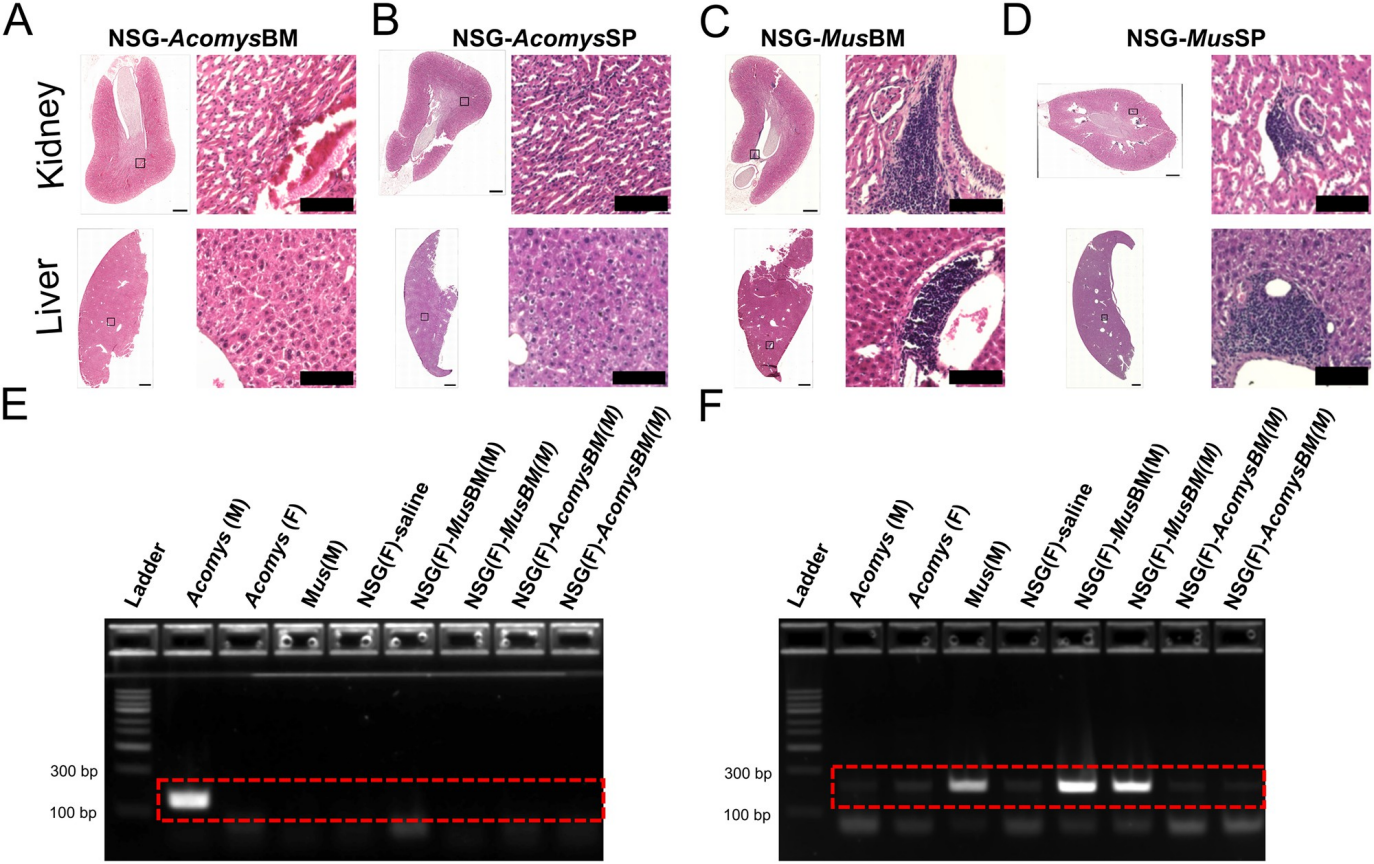
*Acomys* cells fail to engraft and trigger GVHD-like pathology. (A-D) Representative images of H&E-stained sections of kidney (upper panel) and liver (lower panel). Absence of clusters of mononuclear cells in kidney and liver of NSG-*Acomys*BM (A) and NSG-AcomysSP (B) suggesting absence of GVHD-like pathology. The presence of clusters of mononuclear cells in kidney and liver of NSG-*Mus*BM (C) and NSG-*Acomys*BM suggesting occurrence of GVHD-like pathology in NSG-*Mus* chimeras. Scale bars in tiled images 500 μm and enlarged images are100 μm. (E-F) Representative PCR results from DNA obtained from peripheral blood of female recipients show absence of *Acomys* Y-chromosome in female NSG recipients (E) and presence of *Mus* Y-chromosome in female NSG recipients (F).

In addition to spleen and peripheral blood, we evaluated the livers and kidneys from animals that received BM from *Mus* and *Acomys*. In the majority of NSG-*Mus*BM animals, we observed clusters of mononuclear cells in the kidneys and livers (Fig 3C). However, there was no obvious evidence of clusters of mononuclear cells identified in the tissues of NSG-*Acomys*BM animals (Fig 3A). In keeping with numerous previous reports, we considered clustering of mononuclear cells as a sign of a mild GvHD-like response, as GvHD is associated with reconstitution of the hematopoietic system after bone marrow transplant [36]. Next, we wanted to see if mature splenocytes from *Acomys* would mount a GvHD-like response in NSG. To test this, we transferred *Acomys* splenocytes *via* tail-vein into irradiated NSG mice (NSG-*Acomys*SP, n = 3). For controls, we also included NSG-*Mus*SP group (n = 3). However, we did not observe any macroscopic signs of severe GvHD, such as dramatic weight-loss, hair loss and hunched posture, as reported in xenogeneic transplants [37]. Microscopically, GvHD-like pathology was also absent in the kidney and the liver of NSG-*Acomys*SP animals (Fig 3B). Though dramatic weight loss, hair loss, and hunched posture were observed, clusters of mononuclear cells were observed in the kidney and liver (Fig 3D) of NSG-*Mus*SP animals, similar to the fully reconstituted NSG-*Mus*BM chimeras (Fig 3C). The lack of reconstitution of *Acomys* immune system and the absence of GvHD-like pathology, even after the transfer of mature lymphocytes, suggests that transferred cells are not surviving enough to engraft and reconstitute and are being cleared from the periphery. To further confirm this, we screened the female recipients (donor cells were obtained from males) using species-specific Y-chromosome primers and did not detect presence of *Acomys* Y chromosome in all NSG-*Acomys*BM groups (Fig 3E). Successfully reconstituted female NSG mice that received BM from male *Mus* were positive for *Mus* Y chromosome (Fig 3F).

## Discussion

Here in, we sought to create *Acomys*-*Mus* chimeras that would have enabled us to interrogate the role of immune cells from regenerative *Acomys* and their interplay with stromal cells in regeneration and fibrosis. Despite using two xenotransplantation techniques that have been deemed successful in generating chimeras [22, 23, 27, 34], we did not observe engraftment, reconstitution, and differentiation of *Acomys* immune system, nor the development of GvHD-like pathology in NSG mice. The failure to engraft *Acomys* cells is possibly due to complexities of *Acomys* biology rather than technical issues since we confirmed the presence of CD45+ leukocytes, B220+ B cells, CD3+ T cells, and signs of GvHD-like pathology in NSG mice transplanted with both BM and splenocytes.

One possible explanation for the failed engraftment is likely the lack of signals and factors needed for *Acomys* HSCs to engraft, reconstitute, differentiate, and mature under the mouse microenvironment. In adults, HSCs are recruited and maintained primarily in the bone marrow where the microenvironment includes other cell types that secrete cytokines that promote homing, maintenance, proliferation, and differentiation [30]. Many cytokines needed to maintain and regulate HSC function are species-specific [38]. Following allogenic *Mus* transplants where donor and host are likely to share cytokine specificity, we observed successful engraftment and reconstitution. However, in the case of xenogeneic *Acomys* cells, without the specificity for the host cytokines and signaling cues, transplanted cells may have lost their ability to survive and proliferate under a mouse microenvironment leading to cell death. A previous study attempted to restore B cell function by transferring *Acomys* BM into SCID mice and reported the failure to differentiate into B cells [32]. In the same study, the authors also transferred BM from gerbils (*Meriones*), which are phylogenetically closer to *Acomys* than *Mus* [39, 40], and *Meriones* BM also failed to reconstitute [32]. Though SCID mice lack mature T and B cells, their innate immune system and NK cell activity is intact, potentially rejecting the graft from the periphery. However, in our study, we used NSG mice, a severely immunocompromised strain with impaired NK cell activity and defective innate system, which are thought to be more permissive for xenografts. The use of a more severely immunocompromised host in our studies suggests that it is less likely the host is rejecting the transplanted cells and more likely the lack of species-specific cytokines and growth factors impeding the survival and engraftment of *Acomys* cells under the *Mus* microenvironment. Furthermore, though NSG mice support human hematopoiesis, to some extent, NSG hosts are often genetically manipulated to express human cytokines or co-transplanted with fetal liver and thymus to ensure human HSCs receive the necessary signals to further improve the reconstitution and differentiation potential of the human immune system in mice [22, 41–43]. These studies along with our data further indicate the necessity of a conducive environment for xenografts to survive, reconstitute, and differentiate under a mouse microenvironment.

In addition, humanized mice are generated using enriched human HSCs, often derived from umbilical cord blood. In our study, we used whole BM from adult *Acomys* as enrichment of *Acomys* HSCs was not possible due to lack of appropriate cellular and molecular tools, namely a dearth of antibodies that reliably cross-react with *Acomys* immune cells. With the development and validation of *Acomys*-specific reagents and protocols, one could enrich HSCs that would likely increase the chances of establishing *Acomys* hematopoietic system in the mouse environment. Taking one step further, co-transplantation of fetal *Acomys* thymus and liver along with enriched HSCs, similar to BLT approach as seen in the context of humanized mice [43], may further aid in the establishment of *Acomys* hematopoietic system in the NSG host. However, it is important to note that *Acomys* animals and thus raw cell donor populations remain constrained due to long gestational periods, small litter size, and lack of commercial colonies [1, 5].

Though our attempts to engineer *Acomys*-*Mus* chimeras with existing resources and technology were not successful, future attempts may achieve better results upon the development and validation of appropriate reagents and protocols to enrich *Acomys* HSCs and establishment of protocols to co-transplant secondary lymphoid tissues (liver, thymus) or genetically modify mouse to express receptors for *Acomys* cytokines to facilitate the survival and localization of transplanted *Acomys* cells in the mouse host. Our motivation behind generating *Acomys*-*Mus* chimeras was to investigate the causal role of *Acomys* immune cells in regeneration and fibrosis, and these proposed investigations still remain relevant. Specifically, using *Acomyzed* mice, we had plans to investigate the role of *Acomys* immune cells in recovery from several injury models and pro-fibrotic conditions such as biopsy punch of skin [4, 14] and ears [6, 44, 45] and repeated insults of skeletal muscle [7]. In light of the complexities of building *in vivo* chimeras, *in vitro* alternatives are attractive to examine the interaction of immune and stromal cells in regeneration. We and others continue to investigate co-culture testbeds of immune cells from *Acomys* and stromal cells from *Mus* and vice-versa, cell-derived extracellular matrices [46] to study the interaction of immune cells from one species with ECM derived from fibroblasts of another species, and CRISPR based gene editing of cell lines [47] could provide meaningful insights on the mechanistic basis for scar-less regeneration in *Acomys*.
